# Supplementary material for: Antibiotic prescribing practices in community and clinical settings during the COVID-19 pandemic in Nairobi, Kenya
Source: PLOS Glob Public Health. 2024 Apr 25;4(4):e0003046. doi: 10.1371/journal.pgph.0003046 (PMC11045065; doi:10.1371/journal.pgph.0003046)
Supplement: Attachment — Submitted filename: Response to Reviewers.pdf [file pgph.0003046.s003.pdf]

Antibiotic prescribing practices in community and clinical settings during the COVID-19 pandemic in a rapidly urbanising city

Reviewer #1:

Study limitations show weaknesses within a research design that may influence outcomes and conclusions of the research. The limitation mentioned (In the community survey, we extrapolated antibiotic use based on reported sales data, rather than directly tracking antibiotic prescriptions linked to specific diagnoses, making it challenging to determine whether the prescriptions were appropriate or empiric) in this research is very gross. Accordingly, it is difficult to generalize the study finding of community pharmacies where there is no recorded clinical data. It is very difficult to drive findings from sales data for this specific research.

Response: We acknowledge the reviewer's concern that sales data, whether recorded or self-reported by pharmacists as applied in this study, are not ideal proxies for antibiotic use in the absence of clinical records. However, in the context of community pharmacies in low- and middle-income countries where antibiotic use often occurs without prescriptions or diagnoses, and prescribing practices are generally low, our approach aligns with similar studies conducted in similar settings. To emphasize that our conclusion is drawn from information reported by a pharmacist, and the limitations therein, we have revised the discussion to reiterate this point.

It is better to answer the following questions

1. What is the operational definition of suspected COVID 19 infections in community context where there is no clinical data and prescription?

Response: We agree that defining a suspected COVID-19 infection in the community in the absence of confirmed diagnosis or clinical data is ambitious. In this study, our definition was based on what the pharmacists considered to be COVID-19 based on clinical symptoms presented by the patient, or the few patients who had a confirmed diagnosis. However, we acknowledge that these presented symptoms were not always specific to COVID-19, thus increasing the likelihood of false positives and overestimating the weekly number of cases. This approach is similar to other studies studying antibiotic use in LMIC communities:https://www.ncbi.nlm.nih.gov/pmc/articles/PMC9919751/#CR32 and https://www.thelancet.com/journals/lanwpc/article/PIIS2666-6065(22)00035-9/fulltext#seccesectitle0011. We have revised L375-384 in the discussion section to reflect this limitation.

2. From what type of records the researcher got what symptoms are commonly associated with the disease in case of community pharmacies?

Response: Symptoms included in this analysis were reported by pharmacists as those provided by customers when purchasing antibiotics. This symptom checklist pre-loaded on the survey tool was based on the WHO COVID-19 symptom list at the time. It is important to note that pharmacists were not able to confirm COVID-19 diagnoses, and these reported symptoms may not have been specific to COVID-19. This limitation is acknowledged.

Reviewer #2: The article is well organized and very well written. It deals with the antibiotic misuse during the COVID-19 pandemic which impacts the AMR.

The only issue in the manuscript is the number of comorbidities in COVID-19 patients and in those in general wards. Now it is written that only 14% of COVID-19 patients had comorbidities which is not correct.

Authors' response: We thank the reviewer for the correction. L213-214 now reads: Comorbidities were present in 444 (44.8%) of patients in the COVID-19 wards and 215 (29.1%) of patients in the general ward.
