# Supplementary material for: Antibiotic prescribing practices in community and clinical settings during the COVID-19 pandemic in Nairobi, Kenya
Source: PLOS Glob Public Health. 2024 Apr 25;4(4):e0003046. doi: 10.1371/journal.pgph.0003046 (PMC11045065; doi:10.1371/journal.pgph.0003046)
Supplement: Attachment — Submitted filename: Reviewer.docx [file pgph.0003046.s002.docx]

Study limitations show weaknesses within a research design that may influence outcomes and conclusions of the research. The limitation mentioned (In the community survey, we extrapolated antibiotic use based on reported sales data, rather than directly tracking antibiotic prescriptions linked to specific diagnoses, making it challenging to determine whether the prescriptions were appropriate or empiric) in this research is very gross. Accordingly, it is difficult to generalize the study finding of community pharmacies where there is no recorded clinical data. It is very difficult to drive findings from sales data for this specific research.

It is better to answer the following questions

1. What is the operational definition of suspected COVID 19 infection in community context where there is no clinical data and prescription?
2. From what type of records the researcher got what symptoms are commonly associated with the disease in case of community pharmacies?
